# Characterization of ladybird *Henosepilachna vigintioctopunctata* transcriptomes across various life stages

**DOI:** 10.1038/sdata.2018.93

**Published:** 2018-06-05

**Authors:** Qi-Lin Zhang, Feng Wang, Jun Guo, Xian-Yu Deng, Jun-Yuan Chen, Lian-Bing Lin

**Affiliations:** 1 Faculty of Life Science and Technology, Kunming University of Science and Technology, Kunming 650500, China; 2 State Key Laboratory of Pharmaceutical Biotechnology, School of Life Science, Nanjing University, Nanjing 210023, China; 3 LPS, Nanjing Institute of Geology and Paleontology, CAS, Nanjing 210008, China

**Keywords:** RNA sequencing, Entomology, Evolutionary genetics

## Abstract

*Henosepilachna vigintioctopunctata* is a vegetable pest that has spread worldwide. It belongs to the Coccinellidae family, whose members exhibit remarkable diversity, both in terms of their diets and the colored spots that appear on the elytra in the adult stage. Transcriptomic data from *H. vigintioctopunctata* at different life stages would be useful for further investigating the genetic basis of this dietary diversity and the formation of the colored spots in ladybird beetles, as well as revealing the population dynamics of *H. vigintioctopunctata*, which could be useful in pest control. Here, we generated a comprehensive RNA-seq data set (a total of ~24 Gb of clean data) for *H. vigintioctopunctata* by sequencing samples collected at different life stages. We characterized the transcriptomes of each of the four life stages (egg, larva, pupa, adult) and generated a high-coverage pool by combining all the RNA-seq reads. Furthermore, we identified a catalog of simple sequence repeat (SSR) markers. This represents the first study to collect transcriptome data from all life stages of a ladybird beetle.

## Background & Summary

*Henosepilachna vigintioctopunctata* (Coleoptera: Coccinellidae: Epilachninae) belongs to the Coccinellidae family, also known as the ladybird beetles, which contains 90 genera and over 1000 species^1^. This family has been intensively studied, with research areas including the food plasticity, development, phylogeny, population genetics, color polymorphism, and pest control of these beetles^1–4^. In particular, food preferences in the family are highly diverse. Most Coccinellidae species are beneficial to agriculture, acting as predators of aphids, fungal spores, and moth larvae. However, a few of the Coccinellidae species are pest insects^1,3^, eating pollen and even vegetables. Among the pest Coccinellidae species, *H. vigintioctopunctata* is one of the most destructive pests, causing heavy damage to vegetable crops in some countries, principally those belonging to the Solanaceae family (i.e., *Solanum tuberosum*, *S. melongena*, *Lycopersicon esculentum*, and *Lycium* spp.). Adults and larvae feed on the epidermal tissues of leaves and fruits, resulting in considerable economic losses^5^. Extensive studies have been conducted on the ecology and toxicology of *H. vigintioctopunctata*^5,6^. Nevertheless, information on the population genetics, adaptive evolution to different environments, and molecular mechanisms of insecticide resistance in *H. vigintioctopunctata* remains scarce due to the lack of genomic resources, such as genome, transcriptome, and molecular marker (i.e., simple sequence repeat, SSR) sequences. Thus, this is a major impediment to the development of alternative control strategies and new insecticides for this pest.

Some studies have attempted to use comparative genomic analysis to reveal the genetic basis of dietary diversity in mammals and grass carp (*Ctenopharyngodon idellus*)^7–9^; however, these groups are less ideal as models than the Coccinellidae due to their high genetic divergence and artificial dietary interventions. In contrast, the high dietary diversity of the Coccinellidae family, together with their short life cycles, reduced food consumption, and low collection cost, make these beetles the ideal model animal for investigations into the genetic basis of dietary differentiation. In traditional classifications, the Coccinellidae family is composed of eight subfamilies, and their dietary habits can generally be divided into three categories: phytophagous, fungivorous, and carnivorous (Table 1)^10^; thus, information on phytophagous Coccinellidae, which belong entirely to the Epilachninae subfamily, is essential for understanding the dietary evolution of the Coccinellidae family. However, we found that only one phytophagous Coccinellidae species, *Epilachna varivestis*, is currently represented in the NCBI Sequence Read Archive (SRA). Moreover, genomic resources involving the various life stages are lacking in the Coccinellidae family. Therefore, transcriptomic data from *H. vigintioctopunctata* across all life stages will be useful for investigating the genetic basis of dietary diversity among different Coccinellidae groups and the expression patterns of phytophagous-related genes among different life stages.

**Figure 1:**
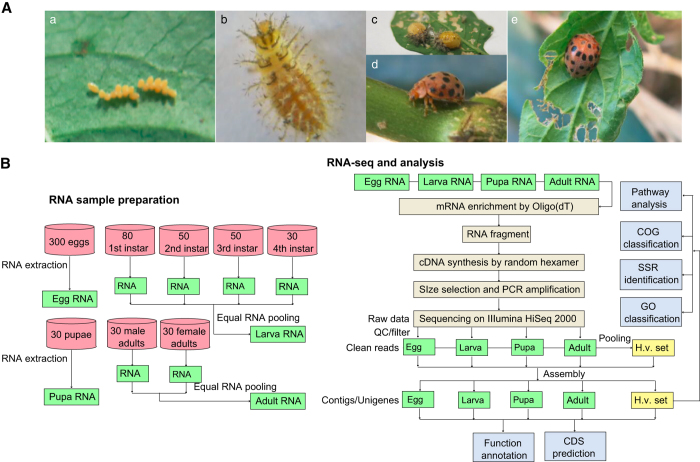
Four life stages of *Henosepilachna vigintioctopunctata* and a flowchart of the experiment. (**a**) eggs, (**b**) larva, (**c**) pupa, (**d**) male adult (**e**), and female adult.

In addition, the contrasting black spots and fringes on the elytra of ladybird beetles often attract considerable attention, and differences in the color or numbers of spots and fringes indicate the extreme morphological and color diversity among the elytra of the Coccinellidae family^1^. A bright coloration is usually employed by predatory ladybird beetles as an aposematic mark for predation and defense, whereas the phytophagous Coccinellidae species trick predators into avoiding them by mimicking this coloration on their elytra. However, the genetic basis underlying the formation of the black spots and fringes is largely unknown. The colored spots and fringes on the elytra are only found in the adult stage (Fig. 1a). Thus, transcriptomic data from *H. vigintioctopunctata* across different life stages will be useful for exploring the molecular mechanisms underlying the formation of the colored spots and fringes, as adult and pre-adult transcriptomes can be compared.

RNA-seq is an efficient, low cost method for the transcriptomic sequencing of non-model animals^11^. Here, the transcriptomes of *H. vigintioctopunctata* from all four life stages were sequenced using RNA-seq technology. *De novo-*assembled unigenes were annotated and extensively analyzed, generating the first deep-sequencing gene catalogs and SSR markers for the Coccinellidae family. The transcriptomes of the four life stages, together with the SSRs derived from the RNA-seq of *H. vigintioctopunctata*, provide a useful resource for studies into pest control and the genetic basis of the high dietary and morphological diversity found in the Coccinellidae family.

## Methods

### Insect materials

*H. vigintioctopunctata* male and female adults were collected from wild *Solanum nigrum* in the garden of the Evo-dev Institute of Nanjing University, Beihai, Guangxi, China (21°25ʹ22.90ʹʹN, 109°08ʹ45.50ʹʹE). The beetles were reared on *S. nigrum* leaves at 27±1 °C, 80±5% relative humidity, and a 12-h light/dark photoperiod cycle in an illumination incubator (Sanyo, Japan) according to methods of Zhou *et al.*^12^ The life cycle of *H. vigintioctopunctata* includes four major life stages: egg, larva, pupa, and adult. These four stages are easily distinguished by appearance (Fig. 1a). Damp filter paper was used to collect eggs after 24 h of oviposition, and eggs were incubated for about 4 days at 27 °C (ref. 12). *H. vigintioctopunctata* has four larval instars, which were identified and confirmed according to methods used in previous studies^12^. After hatching, the insects are considered 1st instar larvae, with a developmental period of approximately 3 days at 27 °C. Subsequently, the larvae molt and then develop into the next instar stage. The developmental periods of the 2nd, 3rd, and 4th instar larvae were approximately 2, 2, and 5 days, respectively, at 27°C. All *H. vigintioctopunctata* larvae used in this study were collected at 10 h post-molting. Recently mated adults were separated and sexed according to their body size and relative location^12^ (the female is bigger than the male and located below the male during mating). Individuals from each of the life stages, including 300 eggs; 80 1st, 50 2nd, 50 3rd, 30 4th instar larvae; 30 pupae; and 30 female and 30 male adults (Fig. 1b), were collected into a plastic tube (5-mL volume) using tweezers with appropriate specifications. All collected samples were initially stored in RNAlater RNA Stabilization Reagent (Qiagen, Germany) and then transferred to –80^o^C for storage prior to analysis.

### RNA extraction

RNA purification of each life stage was performed as described in our previous study^13^. Namely, we homogenized samples in 1 mL of TRIzol (Invitrogen, USA) reagent per 50–100 mg of tissue using a Dounce homogenizer. Next, the homogenized samples were incubated for 5 min at 25–27 °C. Then, RNA extraction was performed following the manufacturer's protocol for TRIzol (Invitrogen). Residual genomic DNA was removed using the RNase-free DNase Set (Qiagen), according to the manufacturer’s instructions. RNA quality was evaluated using a Nanodrop 1000 spectrophotometer (Thermo Scientific, USA). The OD_260/280_ values of each RNA sample was between 1.8 and 2.0, indicating sufficient quality. Next, the total RNA samples from each of the four larval stages was pooled in equal amounts to generate total RNA for the larval stage, and RNA samples from female and male adults were similarly pooled. Finally, the integrity of the total RNA sample from each of the four major life stages (egg, larva, pupa, and adult) was assessed using an Agilent 2100 Bioanalyzer (Agilent Technologies, USA), with an expected RNA integrity number (RIN) threshold of 7.0.

### cDNA library construction, sequencing, assembly, and annotation

Preparation and sequencing of the four libraries were conducted according to standard procedures at the Beijing Genomics Institute (BGI-Shenzhen, China). Library construction was performed using the mRNA-seq sample preparation kit (100 bp×2 paired-end library; Illumina, USA). mRNAs with poly (A) tails were purified from the total RNA using oligo (dT) magnetic beads (Illumina) and were then cleaved into short fragments (200–700 nucleotides) by incubation in fragmentation buffer at 94 °C for 5 min. First-strand cDNA synthesis was conducted using fragmented mRNA templates and reverse transcriptase with random hexamer primers. Second strand cDNA synthesis was then conducted in a reaction system consisting of buffer, dNTPs, RNaseH, and DNA templates. Double-stranded cDNAs were subsequently purified using a QiaQuick PCR Purification Kit (Qiagen). Next, the quality of the cDNA fragments with sequencing adapters was determined using agarose gels. Fragments suitable for downstream analysis were then amplified via PCR to generate the final RNA-seq libraries. The integrity and quality of the four libraries were confirmed using the Agilent 2100 Bioanalyzer (Agilent Technologies). Finally, the constructed cDNA libraries are sequenced on the Illumina HiSeq 2000 platform.

Adaptors, raw reads containing >5% unknown bases (N), and reads showing a mean quality score <20 (Q20 score) were removed using FASTX (http://hannonlab.cshl.edu/fastx_toolkit/) in order to obtain clean read sequences. Next, Deconseq (v0.4.3, http://deconseq.sourceforge.net/) was used to filter out potential reads from other organisms and non-targeted RNAs, such as rRNAs and RNAs from humans, bacteria, and viruses. High-quality clean reads from each library were pooled to generate a high-coverage pooled transcriptome of *H. vigintioctopunctata*. Clean reads from each of the four life stages of *H. vigintioctopunctata* and the pooled transcriptome were used for *de novo* assembly using Trinity software with default parameters^14^. Only unigenes longer than 150 bp were retained for further analyses.

Subsequently, the CD-HIT-EST program in the CD-HIT package (http://www.bioinformatics.org/cd-hit/) was used with a 95% threshold to cluster sequences and eliminate redundancy in the final assembly, retaining the longest unigene as representative of each cluster. To estimate the quality of the assemblies, clean reads were aligned back to the assembled unigenes using Bowtie v1.2.1 (ref. 15). The completeness of each transcriptomic assembly was estimated by comparison with a core set of arthropod genes using BUSCO (Benchmarking Universal Single-Copy Orthologs) v3 software^16^ according to the manual’s recommended default parameters (http://busco.ezlab.org/). This software tests the completeness of assemblies by comparing unigenes with the conserved gene datasets of several closely related species and calculating the percentage of genes that are ‘complete’, ‘fragmented’, and ‘missing’. To obtain annotation information, unigenes were aligned against the NCBI non-redundant (nr) protein and nucleotide (nt), Swiss-Prot, Clusters of Orthologous Groups (COG), and Kyoto Encyclopedia of Genes and Genomes (KEGG) databases using a local blast search (BLASTx or BLASTn from the BLAST package; https://blast.ncbi.nlm.nih.gov/Blast.cgi). For Gene Ontology (GO) annotation of the unigenes, GO assignments were determined by the Blast2GO pipeline^17^. Coding sequences (CDS) were predicted using previously published methods^18,19^ by first aligning unigenes using BLASTx and a threshold E-value of 10^−5^ against protein databases in the following order: nr, Swiss-Prot, KEGG, and COG. Proteins showing the highest scores in the BLAST results were used to determine the CDSs of unigenes, and the coding regions were translated into amino acid sequences using the standard genetic code. Unigene CDSs with no matches to any database were scanned by ESTScan.

### Discovery of SSRs

We used the unigenes assembled in this study to identify new SSR markers through the MicroSAtellite (MISA) tool (http://pgrc.ipk-gatersleben.de/misa/) according to the classification criteria of repeated units and their corresponding length and based on the methods of D’Esposito *et al.*^11^

## Data Records

The project was deposited into the NCBI SRA database (Data Citation 1). The CDS length distribution of the unigenes of *H. vigintioctopunctata* expressed at each of the four different life stages can be accessed at Figshare (Data Citation 2).

## Technical Validation

In order to comprehensively characterize the transcriptome of *H. vigintioctopunctata*, RNA was extracted from samples at each of the four life stages of egg, larva, pupa, and adult. We performed RNA-seq of the four prepared cDNA libraries using a high-throughput sequencing platform. In total, 293,325,912 reads were generated from the four cDNA libraries, from which 247,963,414 clean reads were obtained following further filtering. Summary data for the raw reads generated by the sequencer and the clean reads are listed in Table 2. Clean reads accounted for 81.78–86.32% of the raw reads in the four libraries, and the percentage of raw reads with Q>20 exceeded 98%, which is better than the results of other similar studies^18,20^. These results indicate that the filtering of raw reads was robust.

**Figure 2:**
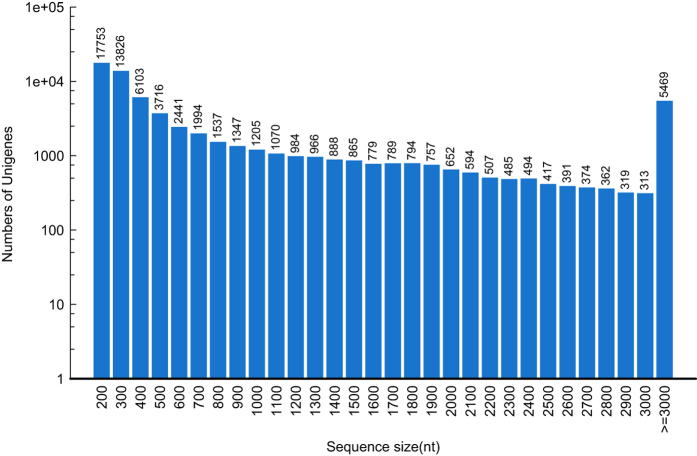
Length distribution of unigenes. The x-axis indicates the lengths of unigenes, and the y-axis indicates the number of unigenes.

**Figure 3:**
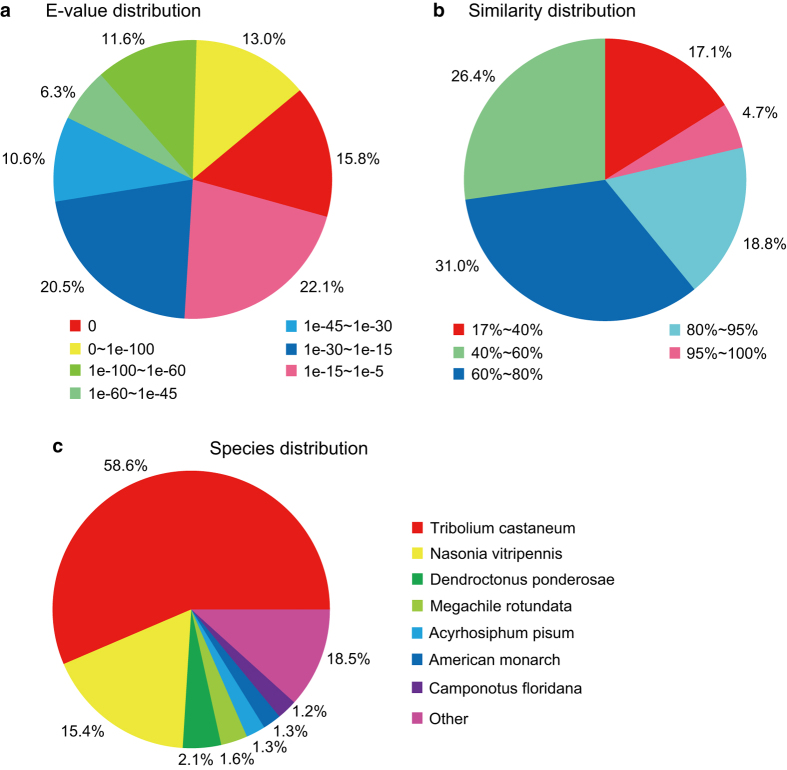
Pie-charts presenting the distribution of E-values, sequence identities, and most closely related insect species resulting from annotation with the nr database.

Together, the clean reads in the pooled transcriptome were assembled into 68,191 unigenes. The total length of all unigenes was 77,193,731 bp, with an average length of 1,132 bp and N50 value of 2,490 bp. Thus, both the mean length and N50 of the unigenes were longer than those previously reported in other related species^18,21^. The assemblies of the pooled and individual life stage samples are summarized in Table 2. As shown in Fig. 2, the unigene length distribution of the pooled transcriptome included 19,474 unigenes that were more than 1,000 bp. The BUSCO analysis, based on 2,675 near-universal single-copy orthologs in arthropod gene sets, showed that 76.2% of unigenes were ‘complete’, 9.3% were ‘fragmented’, and the remaining genes were ‘missing’ in the pooled transcriptome of *H. vigintioctopunctata* (Table 3). By comparing the *H. vigintioctopunctata* assembly with 46 other arthropod lineage assemblies^22^, we observed that the unigenes assembled in this study were of higher quality than the majority of the transcriptome assemblies among arthropods. A total of 41,076 CDSs were predicted from the pooled transcriptome, including 36,680 CDSs that aligned to the protein databases (nr, Swiss-Prot, KEGG, and COG) and 4,396 CDSs predicted by ESTScan. Among the unigenes from the pooled transcriptome, 38,224 (56.05%) were successfully annotated. Among these, a 36926, 18986, 28376, 26232, 13970, and 19080 sequences were annotated based on the nr, nt, Swiss-Prot, KEGG, COG, and GO databases, respectively. Annotation statistics for the unigene sets across the different life stages are provided in Table 4, and detailed annotation information for the unigenes in the pooled transcriptome is provided in Data Citation 2. The E-value distributions showed that 57.4% of the *H. vigintioctopunctata* unigenes from the pooled transcriptome exhibited high homology (E-values <10^−30^) with sequences deposited in the nr protein database, while the remaining 42.6% exhibited weaker homology (10^−30^< E-value<10^−5^) (Fig. 3a). Similarly, 54.5% of unigenes from the pooled transcriptome exhibited >60% similarity to sequences within the nr protein database, while the remaining matches exhibited lower (17%<similarity<60%) similarity scores (Fig. 3b). The distribution of the best species matches indicated that the majority of *H. vigintioctopunctata* unigenes were most similar to homologs in *Tribolium castaneum* (58.6%), followed by *Nasonia vitripennis* (15.4%), and *Dendroctonus ponderosae* (2.1%) (Fig. 3c). *T. castaneum*, the red flour beetle, is the only species closely related to *H. vigintioctopunctata* for which there is a well-annotated, complete genome^23^. Thus, the high homology with the well-annotated *T. castaneum* sequences indicates that the assembly and annotation processes in this study were robust.

**Figure 4:**
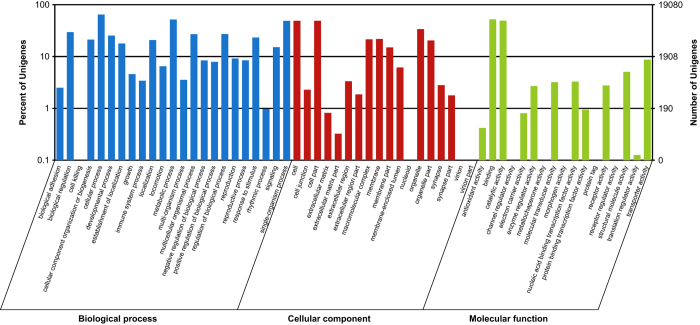
GO classification analysis of unigenes based on biological process, cellular component, and molecular function.

**Figure 5:**
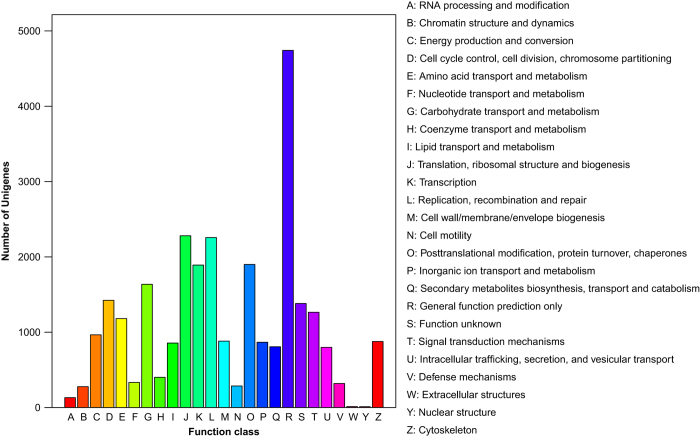
COG functional classification of the *Henosepilachna vigintioctopunctata* transcriptome.

We characterized *H. vigintioctopunctata* unigenes from the pooled transcriptome using the GO, COG, and KEGG databases to conceptually assign transcripts into various groups related to different biological functions. Unigenes annotated with the GO database were classified into three GO categories: biological process (23 GO terms), cellular component (18 GO terms), and molecular function (17 GO terms) (Fig. 4). Using the COG database, 27,779 functional annotations were obtained and grouped into 25 functional categories (Fig. 5). The category containing the largest number of unigenes was the general function prediction only category (4,742 unigenes, 17.07%), followed by translation, ribosomal structure, and biogenesis (2,281 unigenes, 8.21%), and replication, recombination, and repair (2,257 unigenes, 8.12%). Unigenes assigned as RNA processing and modification (131 unigenes, 0.47%), extracellular structures (12 unigenes, 0.04%), and nuclear structure (11 unigenes, 0.04%), exhibited the smallest number of members. To further determine which unigenes were associated with various signaling pathways in *H. vigintioctopunctata*, potential pathways were investigated using all 26,234 sequences annotated with the KEGG database, and sequences were mapped to reference pathway terms in the KEGG database. As a result, a total of 259 KEGG pathways were obtained, with 4,270 unigenes (16.28%) assigned to metabolic pathways, followed by regulation of actin cytoskeleton (1,104 unigenes, 4.21%), and focal adhesion (1,080 unigenes, 4.12%). Pathways involving food digestion and metabolism, insect hormone generation, olfactory transduction, and innate immunity were overrepresented (Data Citation 2). The KEGG terms obtained here showed considerable overlap with those of previous reports on other beetles^24,25^, with over 60% of the terms from this study enriched previously. The comparison of the annotated results obtained in this study with those of other related studies provides technical validation of the current study.

All of the unigenes in the pooled transcriptome were used to discover SSRs. A total of 2,556 SSRs were identified in 2,388 unigenes (Table 5 and Data Citation 2). Among the SSRs detected, the most prevalent SSR type was dinucleotide repeats (51.49%), followed by trinucleotide repeats (36.23%), and mononucleotide repeats (8.10%). The SSR markers identified in the current study will be a valuable resource for investigations into the population genetics, genome organization, and evolutionary rates of *H. vigintioctopunctata* genes. Additionally, because these SSRs were detected at the transcriptional level of the genome, they will be potentially useful for the screening of genes related to insecticide resistance in this pest, but the polymorphic SSRs need to be confirmed via population-level analysis.

## Additional information

**How to cite this article**: Zhang, Q.-L., *et al.* Characterization of ladybird *Henosepilachna vigintioctopunctata* transcriptomes across various life stages. *Sci. Data* 5:180093 doi: 10.1093/sdata.2018.93 (2018).

**Publisher’s note**: Springer Nature remains neutral with regard to jurisdictional claims in published maps and institutional affiliations.

## Supplementary Material



## Figures and Tables

**Table 1:** Dietary habits of different subfamilies in the Coccinellidae family.

**Subfamily**	**Plant**	**Fungus**	**Arthropod**						
			**Aphid**	**Woolly aphid**	**Woolly coccid**	**Scale insect**	**Whitefly**	**Spider mite**	**Other**
Sticholotidinae			**√**	**√**	**√**	**√**	**√**		
Scymninae			**√**	**√**	**√**	**√**	**√**	**√**	
Ortaliinae			**√**	**√**	**√**	**√**	**√**		
Chilocorinae					**√**	**√**			
Hyperaspini			**√**	**√**					
Coccidulinae				**√**	**√**				
Coccinellinae		**√**	**√**	**√**					**√**
Epilachninae	**√**								
The table was cited from the reference^10^.									

**Table 2:** Summary of reads and assembly statistics from transcriptomes of the major life stages of *Henosepilachna vigintioctopunctata*.

**Samples**	**Raw reads**	**Clean reads**	**Clean nucleotides (bp)**	**Q20%**	**GC %**	**Total number of unigenes**	**Total length (bp)**	**Mean length (bp)**	**N50**
Eggs	84,748,254	71,824,146	7,182,414,600	98.60%	39.33%	50,941	49,114,579	964	2,313
Larva	67,645,474	58,391,574	5,839,157,400	98.61%	39.54%	42,442	40,134,316	946	2,041
Pupa	65,858,955	53,852,868	5,385,286,800	98.43%	41.93%	77,574	69,944,184	902	2,000
Adults	75,073,229	63,894,826	6,389,482,600	98.53%	39.46%	43,599	46,045,772	1056	2,261
All (pooling)	293,325,912	247,963,414	24,796,341,400	98.56%	39.51%	68,191	77,193,731	1132	2,490

**Table 3:** Comparison of the *Henosepilachna vigintioctopunctata* assemblies with 2,675 single-copy arthropod orthologs using BUSCO.

**Samples**	**Complete (%)**	**Fragmented (%)**	**Missing (%)**
Eggs	72.1	11.8	16.1
Larva	68.9	13.2	17.9
Pupa	69.4	14.4	16.2
Adults	73.9	10.8	15.3
All (pooling)	76.2	9.3	14.5

**Table 4:** Functional annotations of unigenes from the egg, larva, pupa, adult, and pooled transcriptomes of *Henosepilachna vigintioctopunctata*.

**Sequence file**	**NR**	**NT**	**Swiss-Prot**	**KEGG**	**COG**	**GO**	**ALL**
Eggs	26,201	14,659	21,676	19,704	11,297	13,818	26,913
Larva	21,148	10,890	17,797	15,949	9,563	10,908	21,641
Pupa	21,111	10,442	21,253	19,675	10,727	10,103	22,874
Adults	22,729	11,304	18,886	16,933	10,050	11,523	23,227
All (pooling)	36,926	18,986	28,376	26,232	13,970	19,080	38,224

**Table 5:** Summary of SSRs identified from the *Henosepilachna vigintioctopunctata* transcriptome.

**SSR type**	**Total number**	**Percentage (%)**
Number of unigens containing SSRs	2,388	3.50
Number of identified SSRs	2,556	
Unigenes containing>1 SSR	168	0.25
c (combined SSR)/c*(combined and overlapped SSR)	62	2.43
p1 (mononucleotide repeats)	207	8.10
p2 (dinucleotides repeats)	1,316	51.49
p3 (trinucleotides repeats)	926	36.23
p4 (quadnucleotide repeats)	26	1.02
p5 (pentanucleotide repeats)	11	0.43
p6 (hexanucleotide repeats)	8	0.31
